# 

**Published:** 1925

**Authors:** 


					Vol. XLII. No. 157.
Autumn, 1925,
The Bristol
Medico-Chirurgical Journal
PUBLISHED UNDER THE AUSPICES OF
THE BRISTOL MEDICO-CHIRURGICAL SOCIETY.
Editor: P. WATSON WILLIAMS, M.D.,
WITH WHOM ARK ASSOCIATED
J. LACY FIRTH, M.S., M.D., CAREY COOMBS, M.D.,
E. W. HEY GROVES, M.S., F.R.C.S., B.Sc.
J. A. NIXON, C.M.G., M.D., Assistant Editor.
Editorial Sfcretary: A. L. FLEMMING, M.B., B.Ch.
BRISTOL: J. W. ARRO WSMITtP I^D., 11 QUAY(^jsEET.
nr. :* ?- f\ - yw -
London : J. W. Arrowsmith (London) Ot>h-^p!;I^^er- E&cfoRP Place,
Russell Square.
Price Three Shillings net.

				

